# Supplementary material for: Biomarkers of Coagulation and Inflammation in Dogs after Randomized Administration of 6% Hydroxyethyl Starch 130/0.4 or Hartmann’s Solution
Source: Animals (Basel). 2022 Oct 6;12(19):2691. doi: 10.3390/ani12192691 (PMC9558523; doi:10.3390/ani12192691)
Supplement: Supplementary file 1 [file animals-12-02691-s001.zip › animals-1917624-supplementary.pdf]

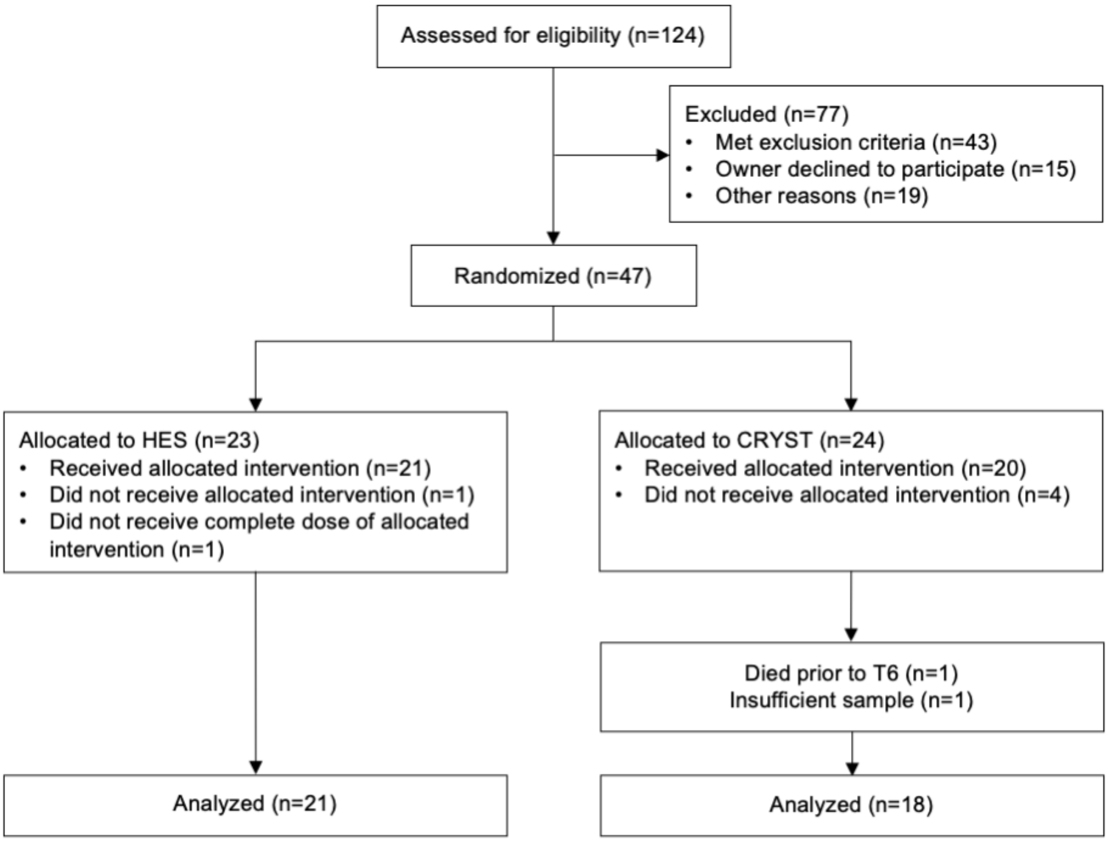

Figure S1. Study flow diagram for inclusion of dogs and randomization to receive 6% hydroxyethyl starch 130/0.4 (HES) or Hartmann's solution (CRYST).

Table S1. Biomarker assay results (median, range, number of samples) in dogs randomized to receive 6% hydroxyethyl starch 130/0.4 (HES) or Hartmann's solution (CRYST).

**Biomarker**	Baseline	T6	T12	T24
**Coagulation biomarkers**				
Prothrombin time (sec)				
HES	8.5 (6.7 – 17.7; n=21)	9.3 (7.3 – 25.5; n=21)	8.9 (7.4 – 22.0; n=18)	9.5 (7.3 – 15.1; n=15)
CRYST	8.2 (7.2 – 10.6; n=18)	8.8 (7.9 – 11.5; n=18)	8.7 (8.1 – 12.5; n=18)	9.3 (7.7 – 15.4; n=16)
Activated partial thromboplastin time (sec)				
HES	17.0 (12.9 – 42.2; n=19)	17.6 (14.1 – 35.8; n=20)	17.6 (11.1 – 34.5; n=18)	19.9 (12.0 – 40.8; n=15)
CRYST	14.9 (9.8 – 21.5; n=17)	14.8 (9.4 – 23.9; n=18)	16.6 (10.4 – 28.7; n=18)	17.7 (9.9 – 50.5; n=16)
Thrombin time (sec)				
HES	7.6 (6.2 – 25.0; n=17)	7.8 (6.4 – 19.9; n=18)	7.5 (6.5 – 19.3; n=18)	7.0 (6.3 – 17.2; n=15)
CRYST	7.6 (5.8 – 10.8; n=16)	7.2 (5.8 – 10.5; n=17)	7.5 (6.0 – 9.9; n=17)	6.9 (6.3 – 11.0; n=16)
Fibrinogen concentration (g/L)				
HES	2.88 (0.59 – 8.52; n=21)	2.55 (0.29 – 8.52; n=21)	3.22 (0.35 – 8.19; n=18)	4.86 (0.51 – 9.66; n=15)
CRYST	3.28 (1.10 – 12.98; n=18)	3.16 (0.97 – 11.11; n=18)	2.82 (1.09 – 11.11; n=18)	3.82 (2.20 – 11.11; n=15)
Factor V activity (%)				
HES	83.5 (9.6 – 167.7; n=17)	74.8 (2.9 – 121.9; n=19)	69.6 (6.5 – 126.8; n=19)	69.6 (19.9 – 148.6; n=15)
CRYST	109.0 (47.5 – 142.7; n=14)	72.2 (19.5 – 121.9; n=18)	56.4 (21.2 – 117.3; n=17)	67.2 (18.4 – 116.9; n=16)
Factor VII activity (%)				
HES	85.2 (18.2 – 234.1; n=18)	70.4 (9.3 – 152.0; n=19)	67.9 (16.4 – 187.1; n=19)	68.7 (22.6 – 203.6; n=15)
CRYST	113.4 (74.8 – 175.3; n=15)	83.6 (53.5 – 140.9; n=18)	73.9 (41.2 – 138.8; n=17)	70.6 (19.3 – 132.7; n=16)
Factor VIII activity (%)				
HES	109.1 (24.5 – 143.2; n=20)	83.7 (21.7 – 122.9; n=20)	84.1 (29.7 – 140.3; n=19)	86.4 (5.9 – 192.3; n=15)
CRYST	124.6 (46.5 – 198.3; n=18)	95.3 (24.8 – 230.5; n=18)	88.3 (24.4 – 278.6; n=17)	90.4 (21.7 – 157.5; n=16)
Factor IX activity (%)				
HES	112.4 (33.0 – 181.2; n=18)	91.0 (23.0 – 129.7; n=19)	90.7 (29.4 – 150.9; n=18)	103.3 (34.0 – 141.4; n=15)
CRYST	118.2 (69.3 – 156.5; n=17)	94.2 (57.1 – 145.5; n=18)	92.3 (55.2 – 215.1; n=17)	96.2 (39.2 – 149.8; n=16)
Factor X activity (%)				
HES	76.2 (19.3 – 146.4; n=17)	69.6 (11.2 – 108.7; n=19)	70.3 (15.0 – 120.9; n=19)	64.3 (23.7 – 118.5; n=15)
CRYST	90.7 (55.7 – 120.0; n=15)	77.0 (46.2 – 93.4; n=17)	63.9 (44.4 – 90.4; n=17)	63.6 (22.3 – 106.7; n=16)

Table S1. cont.

Biomarker	Baseline	T6	T12	T24
**Coagulation biomarkers**				
von Willebrand factor antigen (%)				
HES	124.8 (26.3 – 212.9; n=19)	115.2 (36.2 – 171.7; n=20)	107.8 (31.8 – 181.7; n=18)	134.7 (66.5 – 214.0; n=14)
CRYST	131.9 (51.9 – 217.6; n=18)	115.1 (24.1 – 213.3; n=17)	109.2 (20.4 – 223.8; n=17)	131.6 (24.9 – 220.6; n=15)
Antithrombin activity (%)				
HES	76.3 (52.8 – 110.8; n=19)	69.7 (43.0 – 100.4; n=20)	69.9 (39.5 – 99.2; n=19)	70.8 (39.9 – 114.3; n=15)
CRYST	85.3 (50.6 – 116.1; n=18)	70.8 (5.1 – 95.9; n=17)	64.7 (36.0 – 93.3; n=17)	65.1 (34.6 – 95.3; n=16)
Protein C activity (%)				
HES	86.5 (21.9 – 145.5; n=20)	80.9 (3.1 – 125.9; n=21)	92.3 (3.0 – 128.8; n=19)	108.9 (1.6 – 154.2; n=15)
CRYST	88.9 (49.2 – 180.1; n=18)	84.5 (42.3 – 169.4; n=18)	82.2 (49.3 – 179.5; n=17)	82.9 (34.9 – 182.7; n=16)
**Inflammation biomarkers**				
Interleukin 6 (pg/mL)*				
HES	220.7 (8.6 – 8335.5; n=21)	227.0 (8.6 – 22105.1; n=21)	110.0 (8.6 – 4562.8; n=19)	64.7 (8.6 – 3388.4; n=15)
CRYST	88.9 (8.6 – 1924.8; n=18)	60.9 (8.6 – 3062.0; n=18)	43.4 (8.6 – 2551.3; n=18)	82.5 (8.6 – 3070.8; n=16)
Interleukin 8 (pg/mL)#				
HES	405.4 (113.8 – 3977.3; n=21)	434.6 (8.6 – 139612.3; n=21)	295.9 (8.6 – 29616.0; n=19)	332.0 (8.6 – 6408.5; n=15)
CRYST	633.4 (8.6 – 12863.4; n=18)	494.0 (8.6 – 7896.7; n=18)	360.3 (8.6 – 7363.2; n=18)	364.7 (8.6 – 9228.5; n=16)
Interleukin 10 (pg/mL)$				
HES	25.3 (8.6 – 736.7; n=21)	48.8 (8.6 – 3983.8; n=21)	44.3 (8.6 – 956.6; n=19)	20.3 (8.6 – 185.9; n=15)
CRYST	30.6 (8.6 – 658.6; n=18)	49.9 (8.6 – 347.3; n=18)	18.8 (8.6 – 498.6; n=18)	20.7 (8.6 – 405.3; n=16)
Interleukin 18 (pg/mL)+				
HES	30.9 (8.6 – 666.5; n=21)	28.1 (8.6 – 1391.1; n=21)	35.1 (8.6 – 822.3; n=19)	32.8 (8.6 – 638.3; n=15)
CRYST	8.6 (8.6 – 1836.0; n=18)	15.4 (8.6 – 1047.4; n=18)	26.8 (8.6 – 705.7; n=18)	16.8 (8.6 – 468.3; n=16)
Keratinocyte-derived chemokine (pg/mL)@				
HES	172.1 (18.7 – 2815.6; n=21)	163.3 (8.6 – 3214.9; n=21)	81.1 (8.6 – 3118.5; n=19)	76.2 (8.6 – 587.5; n=15)
CRYST	153.9 (45.3 – 1455.4; n=18)	53.5 (8.6 – 443.7; n=18)	34.0 (8.6 – 721.7; n=18)	58.7 (8.6 – 646.9; n=16)
Monocyte chemoattractant protein-1 (pg/mL)^				
HES	491.8 (8.6 – 17441.2; n=21)	414.0 (8.6 – 25851.9; n=21)	418.3 (8.6 – 10669.7; n=19)	430.8 (8.6 – 3854.6; n=15)
CRYST	544.9 (8.6 – 2978.7; n=18)	306.1 (8.6 – 1186.6; n=18)	101.4 (8.6 – 1561.5; n=18)	372.8 (8.6 – 2136.3; n=16)

* 30 samples were below the assay lower limit of detection of 12.2 pg/mL. # 21 samples were below the assay lower limit of detection of 12.2 pg/mL.
$ 57 samples were below the assay lower limit of detection of 12.2 pg/mL. + 58 samples were below the assay lower limit of detection of 12.2 pg/mL.
@ 14 samples were below the assay lower limit of detection of 12.2 pg/mL. ^ 52 samples were below the assay lower limit of detection of 12.2 pg/mL.

Table S2. Fixed effect P-values for linear mixed effects models analyzing coagulation and inflammation biomarkers over time in dogs randomized to receive 6% hydroxyethyl starch 130/0.4 or Hartmann's solution. These models contained a random effect of dog, nested within treatment, and additional covariates of reason for admission, presence of sepsis, $APPLE_{fast}$ score, and volume of study fluid administered. Significant results ($P<0.05$) are bold.

Biomarker	Time main effect	Treatment main effect	Treatment-by-time interaction effect
Prothrombin time	**0.002**	0.81	0.67
Activated partial thromboplastin time	0.12	**0.039**	0.76
Thrombin time	0.40	0.44	0.61
Fibrinogen concentration	**0.031**	0.91	0.25
Factor V activity	**<0.001**	0.84	0.46
Factor VII activity	**<0.001**	0.59	0.36
Factor VIII activity	**0.005**	0.22	0.18
Factor IX activity	**<0.001**	0.99	0.80
Factor X activity	**<0.001**	0.61	0.46
von Willebrand factor antigen	**0.027**	0.42	0.43
Antithrombin activity	**<0.001**	0.94	0.44
Protein C activity	**<0.001**	0.49	0.07
Interleukin 6*	0.10	0.30	0.24
Interleukin 8*	0.31	0.59	1.00
Interleukin 10*	0.48	0.62	0.36
Interleukin 18*	0.16	0.99	0.67
Keratinocyte-derived chemokine*	**<0.001**	0.11	0.058
Monocyte chemoattractant protein-1*	0.057	0.54	0.95

* data were log transformed for analysis.
